# Understanding Chinese Consumers’ Purchase Resistance in Virtual Live Streaming Rooms: The Role of Negative Anthropomorphism Disconfirmation and Service Guarantees

**DOI:** 10.3390/bs16010099

**Published:** 2026-01-12

**Authors:** Fen Qin, Liting Li, Jie Mi

**Affiliations:** 1 College of Economics and Management, Taiyuan University of Technology, Taiyuan 030024, China; liliting@tyut.edu.cn (L.L.);; 2 Shanxi Key Laboratory of Data Factor Innovation and Economic Decision Analysis, Taiyuan 030600, China

**Keywords:** virtual live streaming rooms, purchase resistance, expectation disconfirmation theory, product uncertainty, service guarantees

## Abstract

Virtual streamers (VSs) and virtual live streaming rooms (VLSRs) are increasingly being utilized in live-streaming commerce. However, consumer purchase resistance in VLSRs remains a salient issue. Grounded in the expectation disconfirmation theory and the product uncertainty perspective, this study adopts a multi-method approach to explore the mechanisms underlying purchase resistance in VLSRs and identify actionable mitigation strategies. Specifically, Study 1 employs a questionnaire survey to examine how negative VS anthropomorphism disconfirmation (NVAD) influences consumer purchase resistance in VLSRs. Study 2 utilizes an experiment to investigate the effectiveness of service guarantees in reducing consumers’ purchase resistance intentions. The results indicate that NVAD impacts consumers purchase resistance in VLSRs via direct dissatisfaction and the chain mediation of perceived product fit uncertainty and dissatisfaction; for consumers with a higher need for interaction with a service employee, the positive relationship between NVAD and dissatisfaction with VLSRs will be stronger; service guarantees can reduce consumers’ resistance. This study enriches the literature on human–AI interaction in VLSRs, consumer resistance behaviors, the expectation disconfirmation theory, product uncertainty, and service guarantees, providing empirical insights for retailers to mitigate consumers purchase resistance in VLSRs.

## 1. Introduction

Live streaming e-commerce has garnered widespread adoption owing to its inherent interactivity, real-time engagement, and authenticity (Xin et al., 2024). Leveraging advancements in digital technologies, particularly artificial intelligence (AI) and three-dimensional (3D) graphics, has attracted growing attention within this commerce model. Virtual live streaming rooms (VLSRs) and virtual streamers (VSs) have become accessible on mainstream Chinese e-commerce platforms, such as Taobao and JD, particularly during the off-peak period. VLSRs combine virtual and real elements through technological empowerment. These constructed environments enable VSs to conduct product introduction and sales activities (Y. Sun et al., 2024). VSs are computer-generated, AI-powered characters who operate without a physical setting on e-commerce platforms and are engineered to emulate the functional roles of human streamers (Gao et al., 2023). The collaboration between VSs and human streamers enables e-retailers to maintain 24/7 live streaming operations at substantially reduced operational costs (Y. Xu & Ruan, 2023).

Despite retailers’ strategic deployment of VLSRs to gain competitive advantages and facilitate sustainable growth, a marked disparity in service quality and gross merchandise value (GMV) persists between VSs and their human counterparts (Peng et al., 2024; Jian, 2025). For instance, the VS I’m Not a Freeloader ran live commerce campaigns for three consecutive months, yet its GMV only exceeded 10 million yuan, less than that of most mid-tier human streamers in the market (Jian, 2025). That is to say, many consumers resist making purchases in VLSRs. Users resistance is widely recognized as a critical barrier to the effective implementation and diffusion of technologies (Ali et al., 2016). Therefore, it is highly necessary to explore the factors that influence consumers’ purchase resistance in VLSRs. However, academic research addressing this phenomenon remains in its nascent stages. Existing studies on the negative impacts of VLSRs have mainly focused on users’ discontinuous behaviors (Y. Chen & Li, 2024). Specifically, these behaviors refer to consumers reducing their level of engagement with VSs or ceasing to watch them, but not to the purchase resistance that occurs after viewing VSs’ live streaming.

Empirically, VSs predominantly rely on preprogrammed dialogues disseminated through voice, static imagery, and prerecorded videos, in contrast to human streamers who deliver dynamic product presentations via real-time video. This standardized mode of content delivery often manifests as mechanical and repetitive, lacking the spontaneity inherent in human interaction (Shao, 2024). As defined by Epley et al. (2007), anthropomorphism refers to the inclination to ascribe human features to non-human. In the context of VLSRs, a critical observation emerges: the current anthropomorphism performance of VSs often falls short of consumers’ anthropomorphism expectations. This misalignment inevitably prompts a question: how does this gap affect consumers’ purchase resistance in VLSRs? To address the question, this study draws on the expectation disconfirmation theory and the perspective of product uncertainty to investigate the influence mechanism of negative VS anthropomorphism disconfirmation (NVAD) on consumers’ purchase resistance. NVAD arises when a virtual streamer’s anthropomorphism performance is lower than consumers’ VS anthropomorphism expectations. Furthermore, the study seeks to propose targeted strategies to mitigate such resistance. Specifically, it raises the following research questions:

RQ1. In terms of streamer attributes, how does NVAD influence consumers’ purchase resistance intention in VLSRs? To what extent do individual differences among consumers moderate this influence mechanism?

RQ2. In terms of product, how do consumers perceived product uncertainty in VLSRs? And how does such uncertainty influence consumers’ negative behaviors in VLSRs?

RQ3. Can the presence of service guarantees in VLSRs effectively mitigate consumers’ purchase resistance in these retail scenarios?

To address these questions, a two-study research design was adopted. Study 1 employed a questionnaire survey targeting Chinese consumers with prior VLSR usage experience, and its primary objective was to unravel the impact of NVAD on consumers’ purchase resistance in VLSRs. Guided by the Cue Utilization Theory, study 2 implemented a between-subjects online experiment with a single-factor (service guarantee: presence vs. absence) design to examine the mitigating effect of service guarantees on consumers’ purchase resistance in VLSRs.

This study contributes to the extant literature in several aspects. First, by identifying the underlying determinants of consumers’ purchase resistance intention in VLSRs, this study strengthens the theoretical framework human-AI interaction in the VLSR context and broadens the scope of resistance literature. Second, this study extends the application of the Expectation Confirmation Theory to the negative outcomes of the emerging VLSRs consumption scenarios. Third, this study contributes to the product uncertainty literature by unpacking the sequential relationships between NVAD, consumers’ perceived uncertainty, and dissatisfaction with VLSRs. Fourth, this study expands the boundaries of service guarantee research and provides actionable strategies for mitigating consumers’ purchase resistance in VLSRs. Fifth, this study indicates that consumers’ evaluation process comprises multiple dimensions and supports the effect of negative emotions in the cognitive-behavior relationship as documented in previous studies (e.g., Ribeiro & Prayag, 2019). Finally, this study offers practical suggestions for e-retailers and VLSR technology providers to increase consumers’ purchase intentions in VLSRs.

## 2. Literature Review

### 2.1. Virtual Live Streaming Rooms (VLSRs) and Virtual Streamers (VSs)

A VLSR is a 24/7 live streaming format, underpinned by the integration of multiple technologies such as AI, deep learning, 3D modeling and speech synthesis. As two core components of VLSRs, virtual streamers (VSs) and live streaming scenes play indispensable roles in its operational mechanism (Y. Sun et al., 2024). VSs are digital avatars with anthropomorphic appearance that emulate human streamers’ behaviors, emotions and conversations (H. Hu & Ma, 2023). Within the live streaming ecosystem, VSs can be primarily categorized into two distinct types (H. Chen et al., 2024): identity-based VSs (e.g., KizunaAI) and service-based VSs (H. Chen et al., 2024). Identity-based VSs focus on their personal image, with a strong emphasis on individuality and uniqueness. They establish a fan economy through sophisticated intellectual property (IP) operation strategies. In contrast, service-oriented VSs focus on delivering professional consumer services within specific scenarios, such as shopping guides and broadcasting (Cui & Liu, 2023). Accordingly, this study focuses on the latter type of VSs in the context of VLSRs.

As summarized in Table 1, existing literature on VLSRs and virtual streamers VSs has documented a dichotomy of findings, encompassing both positive and negative implications. From the positive perspective, scholars have identified several key antecedents that exert significant influences on consumers’ behavioral outcomes, including VSs’ inherent characteristics, linguistic styles, the technical attributes of VLSRs, contextual factors, and product categories (Gao et al., 2023; Wu et al., 2023; Y. Sun et al., 2024). These factors have been shown to shape consumers’ purchase intention, engagement, experiential value and willingness to accept (Gao et al., 2023; Wu et al., 2023; Y. Sun et al., 2024). From the negative perspective, Y. Chen and Li (2024) examined how the violation of VSs’ characteristics influence users’ discontinuance behavior, while Shao (2024) the combined effects of multiple barriers, individual characteristics, and shopping motivations on consumers’ switching intention to VSs.

### 2.2. Purchase Resistance Intention in VLSRs

Resistance is a well-established construct, with user resistance to innovation (Ram & Sheth, 1989), AI systems (Longoni et al., 2019; Zou et al., 2024), and personalized marketing (Cai & Mardani, 2023) all having received academic attention. First proposed by Ram (1987), the innovation resist theory (IRT) provides a framework for understanding why consumers resist novel services and products. In their seminal work, Ram and Sheth (1989) categorize innovation adoption barriers into functional barriers and psychological barriers. Several studies have applied this theory to generate in-depth insights into user resistance toward digital technologies, including mobile payment (Migliore et al., 2022), and chatbots (Chang & Hsiao, 2025). This study focuses on purchase resistance intention in VLSRs, which is defined as the consumers’ intention to refuse purchasing products or services after watching live streams hosted by VSs. This construct is distinct from the mere avoidance of VLSRs or discontinuance behavior, and it has not received sufficient attention in existing literature related to virtual live-streaming. Given that the core focus of IRT in previous studies lies in exploring users’ resistance to technological innovations, rather than consumers’ resistance to purchasing behavior within new channels, this study adopts the expectation disconfirmation theory, instead of IRT, as its core theoretical framework.

### 2.3. Expectation Disconfirmation Theory (ECT) and Negative VS Anthropomorphism Disconfirmation

Expectation disconfirmation theory (Oliver, 1980) was first used to understand consumer satisfaction decisions after buying products or services, and later to be used in information systems research (Hossain & Quaddus, 2012). Expectation disconfirmation theory (ECT) highlights the disparity between expectations and actual experience and includes four major constructs: expectation, performance, confirmation, and satisfaction. According to this theory, if the actual experience is equal to the expectation, it will result in consumers’ confirmation; if the actual experience is lower than the expectation, a negative disconfirmation arises (Oliver, 1980).

Anthropomorphism refers to the inclination to ascribe human features to non-human entities (Epley et al., 2007). Anthropomorphism can manifest in various forms, such as physical appearance, behavior, and mental processes (Miao et al., 2022; H. Chen et al., 2024). Based on the definition of performance and expectation (Lankton & McKnight, 2012), VS anthropomorphism performance refers to consumers’ perceptions of how a VS’s anthropomorphism is manifested, derived from their past experiences. In contrast, VS anthropomorphism expectation refers to consumers’ prior predictions or anticipated beliefs regarding the anthropomorphism of VSs. In the context of VLSRs, the rapid advancement of generative AI has led consumers to hold higher anthropomorphism expectations of VSs that have been endowed with the duties of human streamers. Previous research on VSs has demonstrated that VS anthropomorphism serves as a fundamental psychological mechanism that significantly influences the interactions between consumers and VSs (H. Chen et al., 2024; Y. Sun et al., 2024). However, due to the limitations of technology and cost, a VS’ anthropomorphism performance often fall short of consumers’ expectations in practice, resulting in negative disconfirmation. In view of the above, this study posits a novel construct: Negative VS Anthropomorphism Disconfirmation (NVAD) in the context of VLSRs, representing the assessment made by consumers when a VS’s anthropomorphism performance in practice is lower than their expectations for the VS’s anthropomorphism.

### 2.4. Product Uncertainty

Product uncertainty refers to the buyer’s difficulty in evaluating a product’s characteristics and predicting its future performance, which was first theorized by Dimoka et al. (2012). Hong and Pavlou (2014) classifies product uncertainty manifests in two distinct dimensions: product quality uncertainty (PQU) and product fit uncertainty (PFU). PQU refers to the difficulty for consumers to predict how the product will perform in the real-world, while PFU relates to the challenges in evaluating whether a product matches consumers’ needs (Lu & Chen, 2021). Consumers’ uncertainty about products is recognized as one of the major barriers to online sales (Y. Wang et al., 2021). On e-commerce platforms, perceived information asymmetry and perceived seller opportunism increased perceived uncertainty (Tang et al., 2023), while consumers’ perceived physical characteristic similarity with a broadcaster and perceived value similarity with a broadcaster can reduce product uncertainty either directly or indirectly (Lu & Chen, 2021).

## 3. Research Framework and Hypotheses

**Figure 1:**
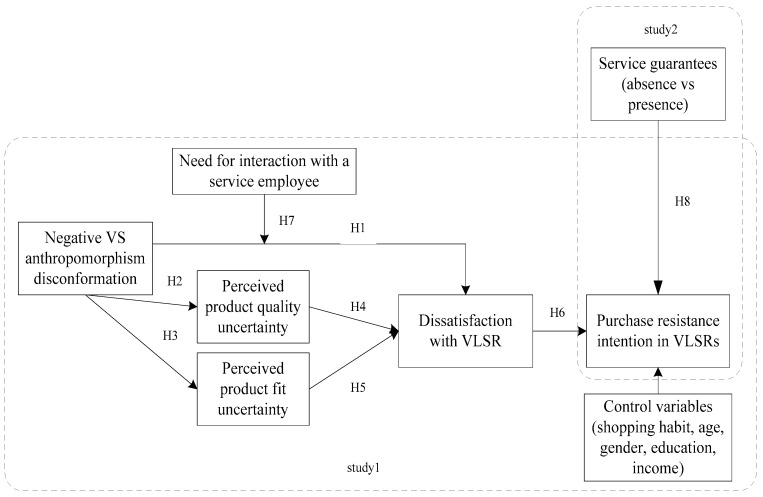
Conceptual model.

The research framework and development of hypotheses in this study are presented in Figure 1.

### 3.1. The Relationship Between Negative VS Anthropomorphism Disconfirmation and Dissatisfaction with VLSRs

Anthropomorphism encompasses multiple dimensions, including human appearances, behaviors and mental states (Epley et al., 2007). VSs’ anthropomorphism affect viewers’ psychological distance toward VLSRs, which subsequently affects their purchase intentions and willingness to accept VSs (Y. Sun et al., 2024; H. Chen et al., 2024). In recent years, generative AI and large language models (LLMs) have experienced rapid development, a trend that is likely to drive consumers to develop higher anthropomorphic expectations for VSs adopting AI technologies. Specifically, consumers anticipate that VSs can exhibit more mentality-related attributes and deliver more personalized service experiences. However, in practice, despite being endowed with numerous human-like cues (e.g., appearance), VSs are unable to understand users’ emotions and experiences, and consequently lack empathy and responsiveness (Y. Chen & Li, 2024). NVAD is caused by the fact that VSs’ anthropomorphism falls short of consumers’ expectation. In line with the satisfaction formation mechanism proposed by the ECT, such negative disconfirmation directly translates into consumers’ dissatisfaction with VLSRs, a construct that reflects viewers’ negative affective responses after engaging with VLSR services (Y. Chen & Li, 2024). Consistent with this theoretical framework, Mostafa et al. (2024) empirically verified a positive correlation between negative virtual chatbot anthropomorphism disconfirmation and dissatisfaction. Therefore, the following hypothesis is proposed:

**H1.**  *Negative VS anthropomorphism disconfirmation is positively associated with consumers’ dissatisfaction with VLSRs.*

### 3.2. The Relationship Between Negative VS Anthropomorphism Disconfirmation and Perceived Product Uncertainty

Product uncertainty is a significant obstacle for consumers to make-decision in online marketplaces (Y. Wang et al., 2021). Within the e-commerce context, this uncertainty arises from three primary factors related to sellers: (1) their inability to perfectly describe the product characteristics through digital interfaces, (2) their lack of unawareness regarding products’ true condition and hidden defects, and (3) their unwillingness to truthfully disclose product quality (Dimoka et al., 2012). Extending this logic to virtual live streaming scenarios, when the anthropomorphism of VSs falls short of consumers’ expectations, consumers perceive that VSs are neither capable of comprehensively presenting product features nor possess an adequate understanding of actual product conditions. Consequently, perceived product uncertainty is triggered among consumers. Furthermore, insufficient VS anthropomorphism relative to viewer expectations may erode consumers’ trust in VSs (H. Chen et al., 2024). Notably, consumers’ perceived trust in streamers exhibits a negative correlation with their perceived product uncertainty (Lu & Chen, 2021). Drawing on the theoretical framework proposed by Hong and Pavlou (2014), the present study categorizes perceived product uncertainty into two dimensions: PPQU and PPFU. Therefore, the following hypotheses are proposed:

**H2.**  *Negative VS anthropomorphism disconfirmation is positively associated with consumers’ perceived product quality uncertainty in VLSRs.*

**H3.**  *Negative VS anthropomorphism disconfirmation is positively associated with consumers’ perceived product fit uncertainty in VLSRs.*

### 3.3. The Relationship Between Perceived Product Uncertainty and Dissatisfaction with VLSRs

Within service industries, uncertainty has been shown to diminish consumers’ valuation of actual performance (Taylor & Baker, 1994). On the e-commerce platforms, a core motivation driving viewers to engage with live streaming content is to acquire product-related information and mitigate their perceived product uncertainty (Lu & Chen, 2021; X. Chen et al., 2023). Thus, if viewers still perceive a high degree of product uncertainty subsequent to engaging with VLSRs, this indicates that the VLSR has not achieved its designed objectives. The cognitive appraisal theory posits that emotion is experienced as a result of the appraisal process (Watson & Spence, 2007). From this theoretical lens, when viewers form unfavorable evaluations of VLSRs (failure to reduce product uncertainty), they are prone to experiencing negative affective states, such as dissatisfaction. Therefore, the following hypotheses are proposed:

**H4.**  *Consumers’ perceived product quality uncertainty in VLSRs is positively associated with their dissatisfaction with VLSRs.*

**H5.**  *Consumers’ perceived product fit uncertainty in VLSRs is positively associated with their dissatisfaction with VLSRs.*

### 3.4. The Relationship Between Dissatisfaction and Purchase Resistance Intention in VLSRs

Extant research has explored the impact of dissatisfaction on various consumer behaviors. For example, dissatisfaction with virtual agent will induce virtual agent avoidance (Mostafa et al., 2024) and discontinuance behavior (Y. Chen & Li, 2024). LaBarbera and Mazursky (1983) found that dissatisfaction with brand X lead consumers to resist purchasing its products. Similarly, Odoom et al. (2019) discovered that when consumers hold negative affective towards a brand, they tend to exhibit an unwillingness to make purchases. Complementing these empirical findings, the cognitive appraisal theory suggests that emotional experiences elicit corresponding behavioral responses (Watson & Spence, 2007). Based on the aforementioned arguments, it can be posited that consumers’ reactions to dissatisfaction in VLSRs align with those observed in other contexts, ultimately prompting purchase resistance within VLSR scenarios. Therefore, the following hypothesis is proposed:

**H6.**  *Consumers’ dissatisfaction with VLSRs is positively associated with their purchase resistance intention in VLSRs.*

### 3.5. Moderating Role of Need for Interaction with a Service Employee

Need for interaction with a service employee (NFI-SE) refers to the importance of human interaction for consumers in service encounters (Dabholkar & Bagozzi, 2002). In their foundational work, Dabholkar and Bagozzi (2002) found that for consumers with strong NFI-SE, the self-service technologies and their features need to be much more humanized and interesting. In VLSRs, VSs serve as the direct service employees that consumers interact with. In this vein, this study anticipates that consumers with a strong NFI-SE will have higher requirements for the anthropomorphism of VSs in VLSRs. If the anthropomorphism of VSs falls short of expectations, consumers with high NFI-SE will consequently exhibit stronger dissatisfaction with the VLSR. Therefore, the following hypothesis is proposed:

**H7.**  *For consumers with higher NFI-SE, the positive relationship between NVAD and dissatisfaction with VLSRs will be stronger.*

### 3.6. The Effect of Service Guarantees

Service guarantees generally include the core services that a firm pledges to provide in order to meet consumer expectations, as well as the remedial actions taken when these commitments are not fulfilled (Hogreve & Gremler, 2009). Beyond this foundational definition, service guarantees embody a comprehensive understanding of service quality standards and service scope boundaries. Functioning not merely as one-way promises, they serve as dynamic strategic tools for proactive service recovery. Empirically, service guarantees significantly enhance both perceived service quality and consumer satisfaction in e-commerce context (Oliveira et al., 2023). This positive effect extends to live streaming commerce domain, where research indicates that service guarantees characterized by moderate commitment levels and flexible terms positively influence consumers’ purchase intentions (Li & Gao, 2024). According to the cue utilization theory, when users view an unknown VLSRs for the first time, their evaluation of a VS’s trustworthiness are predominantly shaped by salient cues present in the VLSRs. In this regard, service guarantees integrated into VLSRs can work as cues signaling the trustworthiness of vendors employing VSs. Trust enhances consumers’ purchase intention in VLSRs (K. Wang et al., 2024). Therefore, the following hypothesis is proposed:

**H8.**  *Service guarantees are negatively associated with consumers’ purchase resistance intention in VLSRs.*

## 4. Methodology and Results

### 4.1. Study 1

#### 4.1.1. Samples and Data Collection

In line with previous research in the context of Chines consumers (Y. Sun et al., 2024), this study used the Taobao as the primary research context. Taobao, which is one of China’s leading e-commerce platform, introduced VLSRs feature in 2020,which has since been widely adopted by major brands like Huaxizi and Dove for product promotion. To empirically test the theoretical framework, this study conducted an online survey via the widely recognized research platform Wenjuanxing (https://www.wjx.cn) (i.e., Shen, 2025) between 1 August and 20 August 2024. The survey implementation followed a rigorous multi-stage process. First, participants were provided with detailed explanations of the research objectives and clear definitions of VLSRs and VSs. To ensure the respondents were familiar with the research context and improve the quality of the questionnaire, a screening question (Have you ever viewed “virtual streamers selling products or services in Taobao virtual live streaming rooms”?) and an open-ended verification question (Please specify the name of the Taobao virtual live streaming room you recently watched) were added. In addition, an attention-check question (requiring participants to select the fixed option “Taobao” from the provided choices) was incorporated to maintain data integrity. Then, respondents were instructed to recall their experience of viewing VLSRs. From an initial pool of 480 responses, this study implemented stringent quality control measures, excluding samples that contained incomplete responses, demonstrated unrealistic completion times (either too short or too long), failed attention-check questions, and showed uniform responses across all items. This rigorous screening process yielded 375 valid responses, representing a 78.1% response efficiency rate. The demographic profile of respondents is presented in Table 2. To assess non-response bias, this study employed a *t*-test to compare demographic variables between the first 20% of participants and the last 20% of participants. The statistical analysis revealed no significant differences between the two groups in terms of age or educational level ($p_{age}=0.834$, $p_{education}=0.917$), indicating that the non-response bias in this study was not serious.

#### 4.1.2. Measurement

Most measurements were adapted from established scales (see Table 3). Specifically, the measurements for “NVAD” and “dissatisfaction with VLSRs” were adapted from Mostafa et al. (2024) by replacing the context of virtual agents with VSs and VLSRs. Both PPFU and PPQU were measured using two items adapted from Lu and Chen (2021). “Need for interaction with a service employee” was measured by a three-item scale adapted from Pichierri and Belk (2026). The dependent variable, “purchase resistance intention in VLSRs”, was measured through a two-item scale combining items adapted from Y. Sun et al. (2024) and Chang and Hsiao (2025). All constructs were scored on a 7-point Likert scale (1 = “totally disagree” and 7 = “totally agree”). To address potential confounding influences, this study incorporated control variables including age, gender, education, and shopping habits. The measurement of shopping habits was adapted from H. Chen et al. (2022).

#### 4.1.3. Common Method Bias

Given the reliance on self-reported data in the study, this study acknowledged the potential presence of common method bias. To this end, prior to data collection, this study designed the questionnaire using clear, unambiguous language. Upon completion of data collection, this study conducted Harman’s single-factor test. The results showed that the sum of squared loadings extracted was approximately 44.709%, which was below the 50% threshold (J. Xu et al., 2014). Subsequently, the correlation coefficients between the constructs were analyzed. As shown in Table 4, the highest correlation coefficient was 0.835, which is below the acceptable maximum of 0.9 (Benitez et al., 2020). These results demonstrate that common method bias poses no significant threat to the internal validity of these findings.

#### 4.1.4. Reliability and Validity

This study employed a partial least squares structural equation modeling (PLS-SEM) approach with SmartPLS 3.0 for data analysis, based on three key considerations: (a) the relatively small sample, (b) the method’s minimal distributional assumptions, and (c) the method’s particular suitability for predictive research models (Hair et al., 2019). This study not only tested existing theories but also added new paths to the theoretical framework.

First, this study deleted one item from the need for interaction scale due to a poor factor loading of <0.3. Other factor loadings exceeded the recommended threshold of 0.708 (Hair et al., 2019), ranging from 0.779 to 0.991 (see Table 5), demonstrating excellent indicator reliability (Hair et al., 2019).

Second, the constructs exhibited strong internal consistency, as evidenced by Cronbach’s alpha values ranging from 0.789 to 0.948 and composite reliability (CR) scores between 0.884 and 0.967. All values surpassed the minimum threshold of 0.7, indicating satisfactory reliability (Fornell & Larcker, 1981; Hair et al., 2011).

Third, the measurement model demonstrated robust convergent validity, with all average variance extracted (AVE) values exceeding the recommended 0.5 threshold (Fornell & Larcker, 1981). Specifically, the AVE values ranged from 0.795 (need for interaction) to 0.936 (purchase resistance intention in VLSRs).

Fourth, this study used the Fornell–Larcker Criterion and the Heterotrait–Monotrait Ratio (HTMT) to establish discriminant validity. The square root of the AVE for each construct exceeded its correlation with other constructs (see Table 4), and no inter-construct correlations surpassed 0.85. All HTMT values remained below the conservative threshold of 0.9 (see Table 6).

The variance inflation factor (VIF) analysis revealed that all formative indicators demonstrated VIF values substantially below the critical threshold of 5 (see Table 5), indicating the absence of multicollinearity concerns in this measurement model.

#### 4.1.5. Structural Model Analysis Results

**Figure 2:**
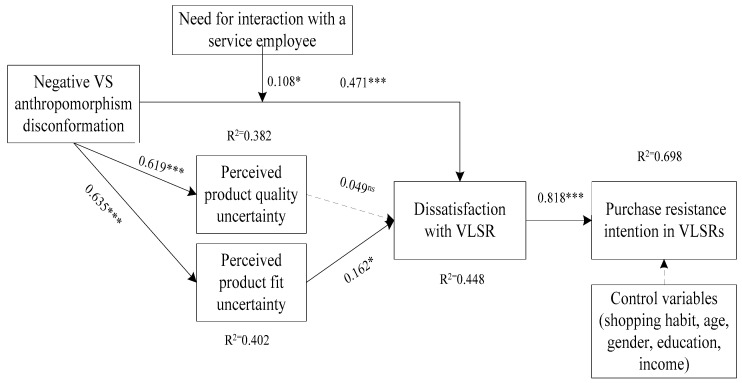
Results of structural model test of study 1. Note(s): ns *p* > 0.1; * *p* < 0.05; *** *p* < 0.001.

To enhance the statistical precision of parameter estimates, this study implemented a non-parametric bootstrapping procedure with 5000 resamples, following established methodological guidelines (Zhao et al., 2010). The adjusted R^2^ values for purchase resistance intention in VLSRs, dissatisfaction with VLSRs, PPQU, and PPFU were 0.698, 0.448, 0.382, and 0.402, respectively(see Figure 2). Based on the criteria proposed by Hair et al. (2011), these values indicate moderate explanatory power for the dependent variables. The results also revealed that the Q^2^ values for purchase resistance intention in VLSRs (0.65), dissatisfaction with VLSRs (0.390) were greater than 0.35, and the Q^2^ values for PPFU (0.335) and PPQU (0.311) were greater than 0. Based on the criteria proposed by Hair et al. (2011), the model had moderate predictive power.

To determine the validity of the hypotheses, this study proceeded to analyze the path coefficients along with their corresponding t-values and *p*-values for every direct pathway (see Table 7). The findings indicated that NVAD was positively associated with consumers’ dissatisfaction with VLSRs (β= 0.471, p<0.001), PPQU ($\beta_{PPQU}$ = 0.619, $p_{PPQU}<0.001$), and PPFU ($\beta_{PPFU}=$ 0.635, $p_{PPFU}<0.001$), supporting H1, H2, and H3. By comparing the coefficients, it could be concluded that the impact of NVAD on PPFU was greater than that on PPQU. Consistent with H5, PPFU led to consumer dissatisfaction with VLSRs (β= 0.162, ρ<0.05), whereas PPQU had no significant impact on consumer dissatisfaction with VLSRs (β= 0.049, ρ>0.1), meaning H4 was not supported. Consumer dissatisfaction with VLSRs strongly explained consumers purchase resistance intention in VLSRs (β= 0.818, ρ<0.001), supporting H6. For consumers with higher NFI, the positive relationship between NVAD and dissatisfaction with VLSRs was stronger (β= 0.108, ρ<0.05). The results also indicated that none of the control variables had a significant impact on consumers purchase resistance intention in VLSRs ($\beta_{age}=0.043,\;p_{age}>0.1;\;\beta_{gender}=-0.026,\;p_{gender}>0.1;\;\beta_{education}=-0.038,$ $p_{education}>0.1;\;\beta_{habit}=-0.030,\;p_{habit}>0.1$).

### 4.2. Study 2

To test the effect of service guarantees, this study conducted a one-factor (presence vs. absence of service guarantees) between-subject design in study 2.

#### 4.2.1. Participants and Design

**Figure 3:**
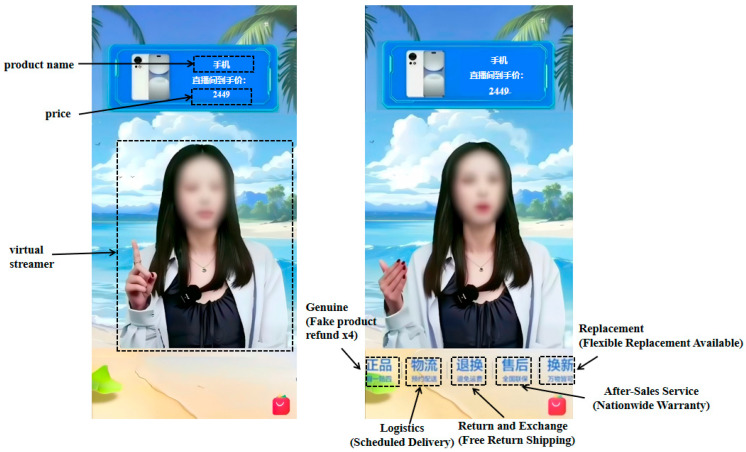
Experimental materials: absence service guarantees (**left**); presence service guarantees (**right**).

In order to ascertain the minimum sample size required for study 2, this study employed the statistical analysis tool G*Power 3.1 (Faul et al., 2009). The analysis indicated that a total sample size of at least 210 participants would be necessary for study 2. Consequently, this study recruited around 280 Chinese participants from www.wjx.cn between 5 January and 15 January 2025. The participants were randomly assigned to one of two experimental conditions. In both conditions, all participants first received an explanation of VSs and VLSRs. Specifically, a VS refers to a digital human driven by technologies such as AI to market and sell goods or services on e-commerce platforms. A VLSR refers to the live broadcast venue where VSs sell products or services. Subsequently, a screening question was incorporated: “Does a VS operated by a human fall within the scope of the VSs defined in this study?” This step served to exclude participants who lacked a clear understanding of the study’s target subject. Participants were then provided with the following instructions: “Imagine you plan to buy a new mobile phone. At 12:30 a.m. one night, you search for ‘mobile phone’ on Taobao and enter a store that indicates it is currently live streaming. After clicking into the store, you see the following scene.” Subsequently, participants viewed a 40 s video clip of a VLSR featuring a female VS selling mobile-phones. To minimize potential confounding influences on the outcomes, the brand names of the VLSR and mobile phone were deliberately obscured. In the treatment condition, the VLSR displayed the following labels: Genuine (Fake product refund x4), Logistics (Scheduled Delivery), Return and Exchange (Free Return Shipping), After-Sales Service (Nationwide Warranty), Replacement (Flexible Replacement Available). The VS, backgrounds, product images, prices, as well as service guarantees were all sourced from real-world scenarios on Taobao. While in the control condition, no service guarantees were displayed. There were no differences between the two conditions in terms of the VS’s image and behavior, the breadth of product information, or product price. Screenshots of the VLSR and VS are presented in Figure 3. While the facial features of the VS have been blurred in this paper to comply with ethical and privacy requirements, no such blurring was performed during the experiment.

#### 4.2.2. Measurement of Study 2

After watching the video, all participants received a prompt stating that this was an AI virtual streamer live stream and were asked to evaluate their perceived VS anthropomorphism and purchase resistance intention in the VLSR based on their feelings while watching the video. Additionally, participants completed a questionnaire regarding the frequency of their shopping through live-streaming rooms, an attention-check question (as used in Study 1), and demographic data (e.g., gender and age). Building on Study 1, this study measured purchase resistance intention in the VLSR using two items: “I do not like to purchase the product through the VLSR” and “I intend to resist purchasing the product through the VLSR.” VS anthropomorphism was measured with five items adapted from Y. Sun et al. (2024). All these items were scored on a 7-point Likert scale (1 = “totally disagree” and 7 = “totally agree”).

#### 4.2.3. Results of Study 2

Prior to formal data analysis, this study first implemented a rigorous data screening process. Questionnaires were excluded if they failed the attention check or had a response time outside the range of 100 to 360 s. After this screening, a total of 252 valid questionnaires were retained, with the control group and the experimental group each consisting of 126 samples. Each participant who submitted a valid questionnaire received a reward of ¥1.5. Among them, 148 (58.7%) were female, and 104 (41.3%) were male. Most participants were between the ages of 21 and 25 (50.8%). The subsequent reliability checks of the scales yield strong Cronbach’s α (purchase resistance intention in the VLSR = 0.920, perceived VS anthropomorphism = 0.875).

**Figure 4:**
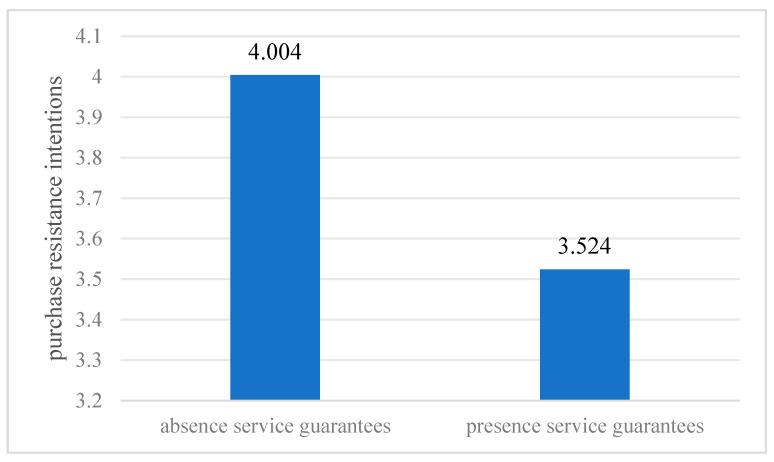
Results in Study2: purchase resistance intention in the VLSR.

For manipulation checks, this study followed Perdue and Summers (1986). Given that the presence of service guarantees was self-evidently different from the absence, no manipulation check seemed necessary (Perdue & Summers, 1986; X. Hu et al., 2010). To test H8, an independent samples *t*-test was conducted on the data from the two groups of participants, and Figure 4 presents the consumers’ purchase resistance intentions in the presence and absence of service guarantees. Results showed that the presence of service guarantee reduced consumers’ purchase resistance intentions from 4.004 to 3.542 (presence service guarantees: M = 3.524, SD = 1.7887 vs. absence service guarantees: M = 4.004, SD = 1.8368, t = 2.012, *p* < 0.05). Therefore, H8 was supported.

## 5. Discussion and Implications

### 5.1. Discussion

Based on expectation disconfirmation theory (Oliver, 1980) and product uncertainty perspective, this study proposes and examines 8 hypotheses, with 7 of them being confirmed.

Firstly, in response to RQ1, this study proposes a novel construct NVAD, which is conceptualized as an application of the ECT. It further reveals two pathways through which NVAD exerts its influence on consumers’ purchase resistance in VLSRs. The first pathway directly triggers consumers’ dissatisfaction with VLSRs, thereby giving rise to their purchase resistance in VLSRs. The second pathway operates through the chain mediation effects of PPPU and dissatisfaction with VLSRs. This study also finds that for consumers with higher NFI, the positive relationship between NVAD and dissatisfaction with VLSRs will be stronger. The results not only validate the expectation confirmation theory, but also complement existing research, which highlights the importance of VSs anthropomorphism in VLSRs (Y. Sun et al., 2024; H. Chen et al., 2024) and the impact of dissatisfaction on consumers’ purchase resistance intention (Chang & Hsiao, 2025).

Secondly, in response to RQ2, this study examines the antecedents of PPQU and PPFU, along with their impact on dissatisfaction with VLSRs and consumers’ purchase resistance intention. The results indicate that NVAD exerts a stronger influence on PPFU than PPQU. Consistent with this finding, C. Sun et al. (2022) also demonstrated that the application of the emerging technology augmented reality exerts a stronger impact on PPFU reduction than on PPQU reduction. Notably, only PPFU significantly leads to consumer dissatisfaction with VLSRs, while the direct effect of PPQU on consumer dissatisfaction with VLSRs is not statistically significant. This implies that, from a product uncertainty perspective, PPFU rather than PPQU serves as the primary driver of consumers’ dissatisfaction with VLSRs. This could be attributed to the fact that the majority of e-retailers utilizing VLSRS boast strong brand reputations, a finding that aligns with the analytical results derived from the VLSR names provided by respondents in study 1. As a powerful endorsement of product quality, brand reputation steers consumers toward prioritizing the fit of products in VLSR scenarios, rather than focusing on product quality per se. Consequently, the adverse impact of perceived product quality uncertainty on consumers’ dissatisfaction with virtual live streaming rooms is substantially mitigated. Future research could conduct further investigations to explore this potential relationship.

Finally, in response to RQ3, this study examines the impact of service guarantees on consumers’ purchase resistance intention in VLSRs, and the results indicate that service guarantees can indeed reduce consumers’ resistance intention.

### 5.2. Theoretical Implications

This study offers several significant contributions to the literature on human-AI interaction, consumer resistance behaviors, ECT, product uncertainty, and service guarantees.

First, by investigating the impact of NVAD on consumers’ purchase resistance intention in VLSRs, this study strengthens the theoretical framework of human-AI interaction and broadens the scope of resistance literature. Most existing research on human-AI interaction in VLSRs has predominantly focused on positive outcomes, such as consumers’ “ willingness to accept” (H. Chen et al., 2024), “purchase intention” (Y. Sun et al., 2024; K. Wang et al., 2024; Gao et al., 2023; H. Hu & Ma, 2023) and experiential value (Wu et al., 2023). Although a handful of studies have touched on negative behaviors, their focus has been limited to discontinuance behaviors (Y. Chen & Li, 2024; Peng et al., 2024). In contrast, this study examines consumers’ purchase resistance behaviors, not merely their avoidance of VLSRs, and analyzes the moderating impact of an individual characteristic, namely the need for interaction with a service employee. This study expands the scope of user resistance from innovation resistance (Ram & Sheth, 1989), AI system resistance (Longoni et al., 2019; Zou et al., 2024), and personalized marketing resistance (Cai & Mardani, 2023) to the novel context of resistance to purchasing via VLSRs. Furthermore, it unpacks the formation mechanism underlying this specific resistance behavior, thereby advancing theoretical insights into user resistance in digital consumption scenarios.

Second, as an application of the ECT, this study proposes a novel concept: NVAD. This study demonstrates that NVAD serves as a predictor of consumers’ perceived product uncertainty, dissatisfaction with VLSRs, and ultimately purchase resistance. This extension not only broadens the theoretical applicability of ECT to the domain of human-AI interaction but also provides a more comprehensive theoretical lens for explaining negative consumer behaviors in emerging consumption scenarios.

Third, this study extends and complements existing research on the impact of product uncertainty in live streaming e-commerce (Lu & Chen, 2021). Prior live streaming commerce research has identified factors that mitigate product uncertainty, such as signal consistency cues and consumers’ perceived similarity with the streamers (X. Chen et al., 2023; Lu & Chen, 2021). Adopting a reverse perspective, this study explores how consumers’ NVAD amplifies their perceived product uncertainty and further investigates the mediating mechanism of perceived PPU and PPFU on consumers’ purchase resistance intention in VLSRs. These findings reveal that NVAD drives perceived product uncertainty; however, only PPFU (not PPQU) translates into consumer dissatisfaction with VLSRs.

Fourth, this study expands the boundary of service guarantees research, which has primarily focused on general e-commerce contexts (Oliveira et al., 2023), by validating its mitigating effect on consumers’ purchase resistance in VLSRs. This empirical confirmation highlights service guarantees as a viable mechanism for reducing consumers’ negative behaviors in VLSRs.

At last, this study indicates that consumers’ evaluation process can consist of multiple aspects (first evaluating the anthropomorphism of VSs, a heuristic cue, and then evaluating product uncertainty, a systematic cue), and supports the effect of negative emotions (e.g., dissatisfaction) in the cognitive-behavior relationship as documented in previous studies (e.g., Ribeiro & Prayag, 2019).

### 5.3. Practical Implications

Against the backdrop of the growing adoption of VLSRs and VSs by e-retailers to reduce broadcasting costs, this study offers a set of actionable practical recommendations for relevant stakeholders.

First, these findings demonstrate that if a VS’s anthropomorphism falls short of consumers’ expectations, this will trigger a cascade of negative consequences (e.g., perceived product uncertainty, VLSR dissatisfaction, and purchase resistance). Consequently, e-retailers may consider prioritize VS anthropomorphism as a core criterion when making VLSR adoption decisions, rather than treating it as a peripheral technical feature.

Second, VLSR technology providers could enhance VSs’ cognitive anthropomorphism and interactive capabilities, while delivering more comprehensive product information through virtual streaming scenarios to mitigate consumers’ perceived product uncertainty. Leveraging advanced technologies to alleviate consumers’ negative disconfirmation toward VSs’ anthropomorphism is particularly critical. For instance, in optimizing VS anthropomorphic presentation, VSs can be programmed with gesture movements synchronized with product introductions; large language models can be employed to generate interactive scripts with a gentle tone tailored to specific interaction contexts. Importantly, VSs can be equipped with real-time interactive functions to cater to the needs of users with high interaction demands.

Third, given that perceived product fit uncertainty directly induces consumer dissatisfaction with VLSRs, whereas perceived product quality uncertainty exerts no significant effect, VLSRs technology providers and adopting e-retailers may consider prioritize addressing PFU over PPU through targeted interventions. For apparel retailers, for example, a dual-system combining “3D virtual fitting + intelligent size recommendation” can be implemented; “scenario-based fit demonstrations” (e.g., deploying VSs of varying heights to showcase clothing try-on effects) can be added; and exclusive “fit consultation” pop-ups can be set up, enabling consumers to connect directly with human consumer service representatives with a single click.

Fourth, since dissatisfaction with VLSRs is a key driver of consumers’ purchase resistance in this context, it is advisable for both technology providers and e-retailers to implement institutional and managerial interventions to mitigate such negative consumer behaviors. A prominent example is the provision of explicit service guarantees in VLSRs, which this study has confirmed to be an effective mechanism for reducing purchase resistance.

## 6. Conclusions

This study aims to examine the formation mechanism of consumers’ resistance to purchase in VLSRs and identify feasible strategies for mitigating such resistance. Based on the ECT and product uncertainty perspective, this study emphasizes the impact of NVAD, perceived product uncertainty and service guarantees on consumers’ resistance intention. The results of empirical research show that dissatisfaction with VLSRs positively affects consumers’ resistance intention. NVAD directly induces consumers’ dissatisfaction with VLSRs, or exerts such an effect indirectly through PPFU. Furthermore, for consumers with higher NFI-SE, the positive relationship between NVAD and dissatisfaction with VLSRs will be stronger. It is worth noting that, service guarantees can indeed reduce consumers’ resistance intention.

This study has a few limitations, which suggest directions for future research. First, the questionnaire data were exclusively collected from Chinese consumers, failing to capture potential cultural variations that may shape consumer responses to VLSRs and VSs in analogous contexts. Cultural dimensions are well-documented to influence consumer decision-making and risk perception. Thus, subsequent research should test these relationships in this study in other cultural context. Second, the data were derived from self-reported questionnaires, future studies could adopt a triangulation approach: combining data from online community observations (e.g., sentiment analysis of VLSR comment sections) and field experiments (e.g., collaborating with e-retailers to track actual purchase resistance behaviors) to collect more ecologically valid data. Third, this study does not provide a satisfactory explanation for why PPQU and PPFU exert differential impacts on consumer dissatisfaction with VLSRs. Future research could consider exploring the effects of factors such as product type and consumers’ level of product knowledge. Fourth, the absence of a manipulation check for service guarantees is defensible in study 2, but it nonetheless represents a minor methodological limitation. Future studies could address this by incorporating a post hoc manipulation check to verify that participants accurately perceived the presence or absence of service guarantees in the experimental conditions. Finally, a further limitation stems from the rapid evolution of AI, which may lead to shifts in consumers’ expectations regarding the anthropomorphism of VSs over time.

## Figures and Tables

**Table 1:** Summary of VLSRs research in e-commerce.

Studies	Theory	Antecedents	Mechanisms	Outcomes	Method
H. Hu and Ma (2023)	language expectancy theory	sensory Language	language expectation violation, VS type	purchase intention	experiment
Y. Xu and Ruan (2023)	pleasure-arousal-dominance model	broadcasters type	social overload, consumers’ senses (pleasure, arousal, dominance)	consumer engagement	survey
Gao et al. (2023)	SOR	likeability, animacy, responsiveness	social presence, telepresence, VSs design (humanoid vs. zoonotic)	purchase intention	survey
K. Wang et al. (2024)	SOR, trust theory	integrity, ability, benevolence, predictability	live room’s social presence, consumers’ trust, streamers type, perceived enjoyment, perceived similarity	purchase intention	mixed-design experiments
Wu et al. (2023)	social response theory	socialness	social presence, communication style, context	experiential value	lab experiments
H. Chen et al. (2024)	social identity theory, construal levels theory	anthropomorphism, product type	psychological distance, trust	willingness to accept	online scenario experiments
Yao et al. (2024)	stereotype content model	linguistic style (social-oriented vs. task-oriented), product type (experience vs. search)	VS type, perceived warmth, perceived competence	purchase intention	online and lab experiments, focus group discussion
Y. Sun et al. (2024)	theory of interactive media effects	technical features (anthropomorphism, media richness)	psychological distance, consumer engagement	purchase intention	survey
Y. Chen and Li (2024)	expectation violations theory, CASA theory	professionalism violation, empathy violation, responsiveness violation	distrust, dissatisfaction	discontinuance behavior	survey
Shao (2024)	innovation resist theory, shopping motivation theory, personality theory	usage barrier, value barrier, risk barrier, image barrier, traditional barrier, utilitarian motivation, hedonic motivation, inertia, affinity for human–computer interactivity		switching intention	NCA, ANNs, fsQCA
Peng et al. (2024)	expectancy disconfirmation theory, stressor-strain-outcome model	information failure, functional failure, system failure, interaction failure	livestreaming platform type, disappointment, emotional exhaustion	discontinuance behavior	survey
This study	expectation disconfirmation theory, product uncertainty theory	negative VS anthropomorphism disconfirmation, perceived product quality uncertainty, perceived product fit uncertainty	need for interaction, dissatisfaction with VLSRs,	purchase resistance intention in VLSR	survey

**Table 2:** Descriptive statistics of samples of study 1 (N = 375).

Category		Frequency	Percentage
Gender	1. Male	210	56%
	2. Female	165	44%
Age	1. 18–25	140	37.3%
	2. 26–30	145	38.7%
	3. 31–40	76	20.3%
	4. 41–50	12	3.2%
	5. ≥50	2	0.5%
Education	1. Senior High school and below	33	8.8%
	2. College	111	29.6%
	3. University	209	55.7%
	4. Graduate degree and above	22	5.9%

**Table 3:** Construct definitions and items.

Construct	Items	Reference
Negative VS anthropomorphism disconfirmation(NVAD)	NVAD1. Compared to my expectations, the VS’s mind is lower than expected. NVAD2. Compared to my expectations, the VS’s consciousness is lower than expected. NVAD3. Compared to my expectations, the VS’s free will is lower than expected. NVAD4. Compared to my expectations, the VS’s emotions are lower than expected. NVAD5. Compared to my expectations, the VS’s intentions are lower than expected.	Adapted from Mostafa et al. (2024)
Perceived Product Quality Uncertainty (PPQU)	PPQU1. I am concerned that the product will look different in real life from how it was described in the e-store. PPQU2. Based on the VS’s recommendations, I doubt that the product’s functions will meet my performance expectations.	Lu and Chen (2021)
Perceived Product Fit Uncertainty (PPFU)	PPFU1. I have reservations about whether the products suggested by the VS will adequately fulfill my needs. PPFU2. The product recommendations provided by the VS do little to alleviate my concerns regarding its suitability for me.	Lu and Chen (2021)
Dissatisfaction with VLSRs (DV)	DV1. I feel dissatisfied with my overall experience of watching a VLSR. DV2. I am not delighted about my overall experience of watching a VLSR. DV3. I feel the overall experience of watching a VLSR is boring.	Adapted from Mostafa et al. (2024)
Need for Interaction with a service employee (NFI)	NFI1. I like interacting with the person who provides the service. NFI2. Human contact in the provision of services makes the process enjoyable for me. NFI3. Personal attention from the streamer is very important to me. *	Adapted from Pichierri and Belk (2026)
Purchase resistance intention in VLSRs (RP)	RP1. I do not like to purchase products or services through VLSRs. RP2. I intend to resist purchasing products or services through VLSRs.	Adapted from Chang and Hsiao (2025) and Y. Sun et al. (2024)
Habit (HAB)	HAB1. Purchasing items through live streaming has become an instinctive habit for me. HAB2. Buying products through live streaming feels like second nature to me. HAB3. Whenever I consider making a purchase, turning to live streaming is my go-to option.	H. Chen et al. (2022)

Note: * = deleted item.

**Table 4:** Fornell–Larcker Criterion of study 1.

Construct	NVAD	PPQU	PPFU	DV	RP	NFI
NVAD	0.909					
PPQU	0.619	0.909				
PPFU	0.635	0.681	0.921			
DV	0.640	0.471	0.524	0.939		
RP	0.578	0.402	0.441	0.835	0.968	
NFI	0.018	0.071	0.035	−0.086	−0.083	0.891

**Table 5:** Results of reliability and validity tests for variables of study 1.

Construct	Item	Factor Loading	VIF	Cronbach’s Alpha	CR	AVE
Negative VS anthropomorphism disconfirmation (NVAD)	NVAD1	0.913	3.918	0.948	0.960	0.827
	NVAD2	0.905	3.760			
	NVAD3	0.909	3.852			
	NVAD4	0.906	3.833			
	NVAD5	0.913	3.795			
Perceived product quality uncertainty (PPQU)	PPQU1	0.892	1.740	0.789	0.905	0.826
	PPQU2	0.925	1.740			
Perceived product fit Uncertainty (PPFU)	PPFU1	0.913	1.951	0.822	0.918	0.849
	PPFU2	0.929	1.951			
Dissatisfaction with VLSRs (DV)	DV1	0.930	3.493	0.933	0.957	0.882
	DV2	0.945	4.315			
	DV3	0.941	4.031			
Purchase resistance intention in VLSRs (RP)	RP1	0.967	4.189	0.932	0.967	0.936
	RP2	0.968	4.189			
Need for interaction with a service employee (NFI)	NFI1	0.779	1.911	0.817	0.884	0.795
	NFI2	0.991	1.911			

**Table 6:** HTMT test results of study 2.

Construct	NVAD	PPQU	PPFU	DV	RP
PPQU	0.715				
PPFU	0.720	0.845			
DV	0.680	0.547	0.598		
RP	0.614	0.468	0.502	0.895	
NFI	0.076	0.117	0.077	0.072	0.067

**Table 7:** Hypotheses results.

Hypotheses	Path	Path Coefficients	T-Statistics	*p* Values	Decision
H1	NVAD → DV(+)	0.471	8.357	0.000	Supported
H2	NVAD → PPQU(+)	0.619	13.702	0.000	Supported
H3	NVAD → PPFU(+)	0.635	14.290	0.000	Supported
H4	PPQU → DV(+)	0.049	0.892	0.372	Not Supported
H5	PPFU → DV(+)	0.162	2.477	0.013	Supported
H6	DV → RP(+)	0.818	32.114	0.000	Supported
H7	NFI moderate NVAD → DV(+)	0.108	2.003	0.045	Supported

## Data Availability

The data presented in this study are available on request from the corresponding author.
